# Ostéosynthèse des fractures des métacarpiens et des phalanges de la main par mini plaque: à propos de 12 cas

**DOI:** 10.11604/pamj.2016.24.224.8325

**Published:** 2016-07-12

**Authors:** Erraji Moncef, Derfoufi Abdelhafid, Kharraji Abdessamad, Agoumi Omar, Abdeljaouad Najib, Daoudi Abdelkrim, Yacoubi Hicham

**Affiliations:** 1 Unité de Chirurgie Orthopédique et Traumatologique, Centre Hospitalier d’Oujda, Maroc

**Keywords:** Métacarpe, phalange, main, mini plaques, Metacarpus, phalange, hand, mini plates

## Abstract

Le traitement des fractures instables des métacarpes et des phalanges reste un objet de controverse. Peu de séries ont été rapportées dans la littérature, rendant leur analyse difficile. Nous rapportons une étude rétrospective comportant 12 patients, opérés par cette technique, ayant eu des fractures déplacées des métacarpes ou des phalanges, sur une période de deux ans. Les résultats globaux ont été bons dans 75% des cas, moyenne dans 16,5% des cas et mauvais dans 8,5% des cas. La stabilité du montage par mini plaques des fractures instables des métacarpiens et des phalanges ont permis une mobilisation précoce des articulations de la main, évitant ainsi la raideur.

## Abstract (en)

Treatment of unstable fractures of metacarpals and phalanges remains subject of controversy. Few series have been reported in the literature, making their analysis difficult. We provide a retrospective study involving 12 patients, operated on by this technique for displaced fractures of the metacarpals and phalanges, over a two year period. The final overall results were good in 75% of the cases, average in 16,5% of the cases and bad in 8,5% of the cases. Stability of mini plates in unstable fractures of the metacarpals and phalanges allowed early mobilization of hand joints, thus avoiding stiffness.

## Introduction

Les fractures des métacarpiens et des phalanges de la main sont des lésions très fréquentes [1, 2]. L’utilisation des mini-plaques pourraient remettre en question le dogme de l’immobilisation postopératoire des fractures de la main. La stabilité de l’ostéosynthèse obtenue par ces dispositifs autorise une mobilisation post opératoire immédiate, permettant une récupération fonctionnelle précoce de la main et une diminution de la durée de l’arrêt de travail [2, 3]. L’objectif de ce travail rétrospectif est de rapporter les résultats clinico-radiologiques de 12 patients et d’analyser les complications d’une série homogène des fractures des métacarpiens et des phalanges de la main ostéosynthésées par mini plaques.

## Méthodes

La série: il y avait six fractures de métacarpiens et six fractures des phalanges (4 fractures de la phalange proximale et 2 cas de la 2^ème^ phalange), chez 12 patients âgés de 18 ans ou plus ont été incluses dans l'étude rétrospective menée au cours de la période 2011-2013 au CHU d’Oujda service de traumato-orthopédie. Les fractures inclues dans cette étude, sont les fracture irréductibles, les fracture déplacé transversalement, oblique court ou oblique long et les fractures articulaire du condyle avec atteinte d’une surface articulaire > 25% [4], par contre les fractures articulaires de la base du pouce, les fractures ouvertes et les fractures sur os ostéoporotiques sont exclues de cette étude.

**Figure 1:**
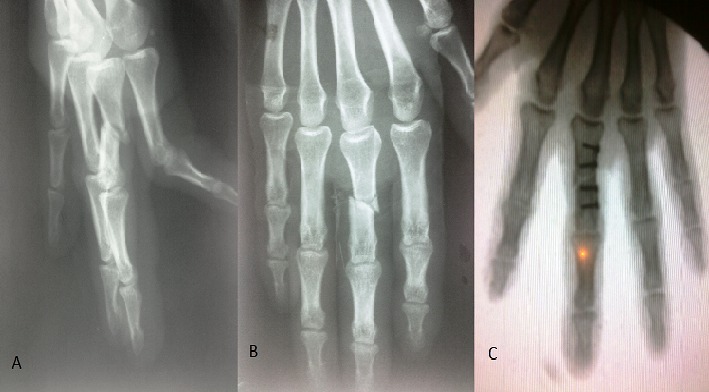
A,B) fracture de la phalange proximale du 3^ème^ doigt (vue de face et de profil); C) après stabilisation par mini plaque

**Technique opératoire:** tous les patients ont été opérés sous anesthésie locorégionale et garrot pneumatique par un opérateur senior et un résident. Une voie d’abord dorsale et dorso-latérale ont été pratiqués respectivement pour les fractures métacarpiennes et les fractures phalangiennes. La synthèse comprendra 2 vis corticales en amont et 2 vis en aval du foyer de fracture. On a utilisé des mini vis de diamètre 2,7 mm ou 2,0 mm pour les fractures métacarpiennes spiroides ou oblique long et pour les fractures des phalanges des mini vis de diamètre 2,0 mm ou 1,5 mm. Une mini plaque droite de 2,7 mm ou 2,0 mm a été utilisé pour la fracture de la diaphyse métacarpienne, ainsi pour les fractures du col ou de la tête on a utilisé une plaque de 2mm en T ou en L, les fractures des phalanges proximales, on a utilisé des mini plaques de 2,0 mm ou 1,5 mm (Figure 1). Une immobilisation post opératoire a été réalisée chez 3 patients et la mobilisation active a été encouragée dès la levée de l’anesthésie locorégionale, le soir même de l’intervention chirurgicale. Les patients ont été revus à la deuxième semaine post opératoire pour ablation des fils, contrôle radiographique et prescription de rééducation le cas échéant, puis régulièrement jusqu’au dernier recul.

**Critères d’analyse des résultats:** les résultats de notre série ont été analysés sur la base des critères subjectifs et objectifs: Les critères subjectifs étaient la douleur mesurée sur une échelle visuelle verbale (EVS: 0 = pas de douleur, 1 = douleur faible, 2 = douleur modéré, 3 = douleur intense, 4 = douleur extrêmement intense) et le score fonctionnel du quick DASH. Les critères objectifs étaient cliniques et radiologiques. L’évaluation clinique est établie selon les critères de la société américaine de la chirurgie de la main par la mesure de la mobilité active totale qui est définie: TAM par le total du mouvement active des 3 articulations (MP, IPP et IPD). (TAM normale pour les doigts = 260°) [2–5], et la durée de l’arrêt de travail exprimée en semaines [2] (Tableau 1). Les critères radiologiques étaient évalués sur des radiographies de face, profil et trois quarts en préopératoire, en postopératoire immédiat, au dernier recul afin d’analyser une perte de réduction et la consolidation osseuse. Des complications ont été recherchées: la migration du matériel, une infection, une lésion nerveuse, un cal vicieux et une gêne esthétique.

**Tableau 1:** Critères objectifs

Résultat	Bon	Moyen	Médiocre
TAM	TAM ≥ 210 °	TAM entre 180° - 210°	TAM <180°

## Résultats

**Figure 2:**
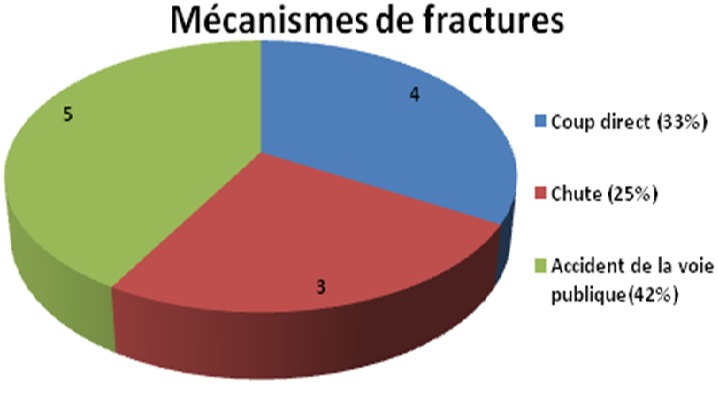
Mécanismes des fractures

**Figure 3:**
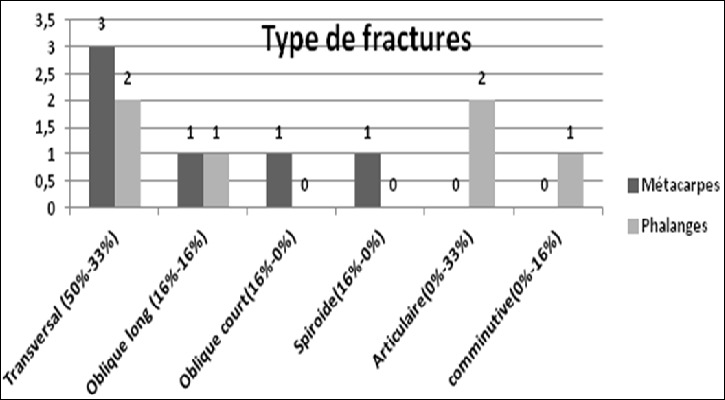
Type des fractures

**Figure 4:**
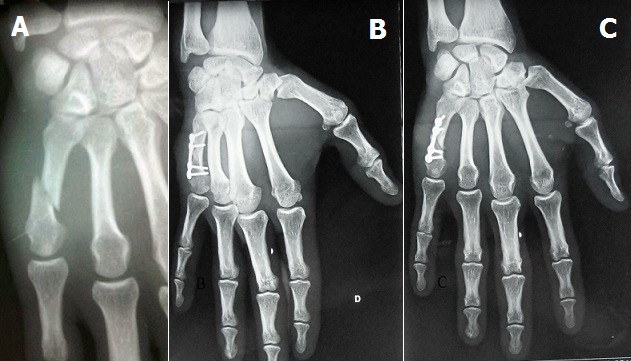
A) fracture à trait oblique du 5^ème^ métacarpe; B) après réduction par mini plaque; C) évolution à 1 mois

**Figure 5:**
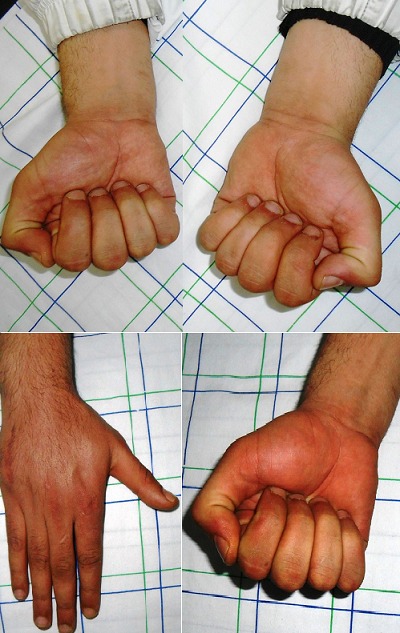
Évaluation clinque chez un patient opéré pour fracture du 5^ème^ metacarpe avec mise en place d’une mini plaque

**Figure 6:**
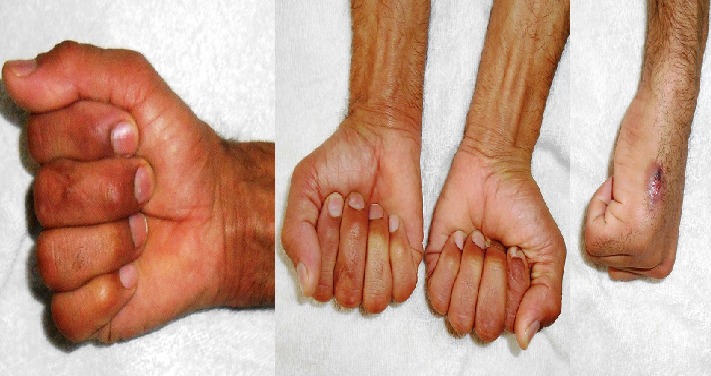
Patient traité pour fracture diaphysaire du M5, l’évolution a été marqué par une infection superficielle de la plaie

**Figure 7:**
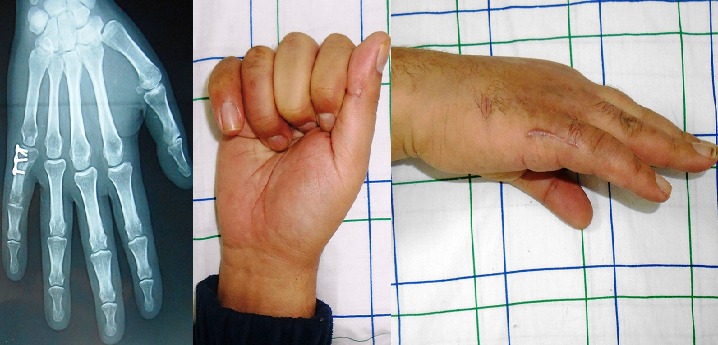
Un patient présentant une raideur digitale suite à une fracture comminutive de la base du P1 du 5^ème^doigt

Tous nos malades ont été de sexe masculin. L'âge moyen était de 26 et 4 mois, la main droite a été impliquée dans 9 cas. Le mécanisme était comme suivants 4 cas suite à une chute, coup directe chez 3 cas et 5 cas suite à un accident de la circulation (Figure 2). Les types de fractures été comme suivants (Figure 3): les fractures à trait transversal ont été observé dans 5 cas; une fracture oblique long dans 1 cas (Figure 4), oblique court dans 2 cas; 1 cas de fracture spiroide, deux fracture articulaire et 1 cas avec fracture comminutive. La durée moyenne de la chirurgie été 46 minutes (extrêmes 35-70 minutes) (Tableau 2). 10 fractures ont été fixé par mini plaque; 3 par ostéosynthèse mixte mini plaque plus mini vis. Les résultats étaient bons (TAM = 210°) dans 9 cas (Figure 5), moyenne dans deux cas (TAM entre 180°-210) et médiocre (TAM < 180°) a été observée dans un seul cas (Tableau 3). Le score fonctionnel du quick DASH avait une valeur moyenne de 16/55. Trois patients avaient des douleurs au repos et deux autres à l’effort.la durée moyenne de consolidation était 11 semaines avec des extrêmes de 8-18 semaines (Tableau 4). 3 cas présentaient des complications jugées mineures: une infection superficielle traité par antibiothérapie (Figure 6) et deux cas de gêne esthétiques suite à une cal osseuse proéminente. Les fractures présentaient des complications jugées majeures: un cas de déformation résiduelle (angulation> 10°) lié à une fracture comminutive et un cas présentant une raideur digitale (TAM150) (Figure 7) (Tableau 5).

**Tableau 2:** Données épidémiologiques

	Métacarpes	Phalanges
**Nombres de patients**	6	6
**Nombres de fractures**	7	6
**Types des fractures**	T: 50% Ob: 32% S: 16%	C: 16%; A: 33%
**Sexes M /F**	6/0	6/0
**Age**	23	27
**Le temps de la chirurgie/jour**	4.25 jr	5 jr
**La durée opératoire/min**	42min	52min

Abréviation: T: transversal; Ob: oblique; S: spiroide; C: comminutive A: articulaire

**Tableau 3:** Résultats des amplitudes articulaires

Catégories des fractures	MP	IPP	IPD
**Normal**	85°	110°	65°
**métacarpe**	76°	102°	62°
**Phalange proximal**	85°	96°	58°
**2^ème^ phalange**	78°	90°	60°

**Tableau 4:** Résultats subjectifs

Phalanges			
Patients	Douleur (EVS)	Fonction (quick DASH/55)	Durée AT(S)
1	0	12	8
2	2	14	5
3	1	19	6
4	NP	NP	NP
5	1	23	5
6	2	13	4
Métacarpes			
7	0	15	5
8	1	17	5
9	1	13	7
10	2	12	5
11	1	13	6
12	2	25	9

EVA: échelle visuelle verbale; Quick DASH: disability arm shoulder hand; SP: sans profession; NP: non précisé

**Tableau 5:** Complication liée à l’ostéosynthèse

Complication	Mini plaque et mini vis
Infection	1
Démontage du matériel	0
Raideur	1
Pseudarthrose	0
Gène esthétique	2
Déformation résiduelle	1

## Discussion

L’ostéosynthèse des fractures de main fait appel à 2 principales techniques: l’embrochage à foyer fermé et l’ostéosynthèse par mini plaque. James rapporte dans sa série que 77% des fractures instables de phalanges traités par embrochage avaient des résultats globaux médiocres et conclue que ce type d’ostéosynthèse reste moins stable avec un contrôle de la rotation trop limité [6–8]. Une étude portant sur 52 fractures extra articulaires des métacarpiens traitées par embrochage, ont été comparés avec ceux des résultats des mini plaques, avec aucune différence significative sur le plans cliniques pour les deux groupes, mais les patients traités par embrochage ont eu une perte de la réduction, avec migration de la broche dans l´articulation métacarpo-phalangienne et nécessitant par la suite une chirurgie secondaire pour l’ablation du matériel [9]. Dans notre série, l’utilisation du mini plaques a permis d’obtenir une réduction anatomique avec un montage qui est suffisamment rigide afin de permettre une mobilisation précoce des articulations adjacentes, ce qui explique que nos résultats fonctionnels évalués par la TAM étaient meilleurs. On a constaté que les patients qui n’ont pas eu une immobilisation complémentaire avaient des résultats fonctionnels très satisfaisants avec une TAM plus de 190°, contrairement à ceux qui ont eu une immobilisation pendant 3 semaine avec comme résultat une TAM de moins de 150°. La majorité de nos patients ont bénéficiés d’une rééducation comportant un massage assouplissant avec un drainage lymphatique manuel pour la mise en confiance du patient pendant les premiers jours. La récupération des amplitudes articulaires et de la force musculaire, sont faite par le travail analytique puis un travail global au cours de la 2ème semaine. Enfin le travail proprioceptif par des exercices globaux sollicitant la main, le poignet est pratiqué pendant les dernières séances pour avoir une main fonctionnelle. De nombreuses études dans la littérature ont montré une supériorité biomécanique des mini-plaques par rapport à d´autres moyens d’ostéosynthèse, cela est confirmé par une étude réalisée par Fyfe et Mason [7] qui a objectivé la rigidité et la stabilité du montage par mini plaque par rapport à l’embrochage qui est plus adapté pour les fractures transversales. Plusieurs études ont rapporté des résultats satisfaisants lors de l’utilisation des mini plaques [8, 10, 11]. Les résultats globaux de notre étude étaient similaires à celles de la série d’Agarwal [8], portant sur 20 fractures de la main traitées par mini plaque. Quatre cas dans notre série avaient des mini vis et des mini plaques trop volumineuses sans retentissement sur les amplitudes articulaires de la main. L’application de la plaques sur la face dorsale des métacarpiens n'a pas eu une répercussion sur l’appareil extenseur des tendons. Nous tenons à souligner que la dissection chirurgicale doit être méticuleuse, en évitant tout traumatisme des parties molles et un déperiostage excessive.

## Conclusion

Bien que l’ostéosynthèse par une mini plaque est une option raisonnable pour le traitement des fractures instables, il faut cependant rester toujours vigilant dans la sélection des patients et l’abord chirurgical. Un bon suivi clinique et radiologique avec un acte chirurgical bien codifié ainsi qu’une dissection minutieuse sont les clés pour obtenir de bons résultats fonctionnelle avec moins de complication.

### Etat des connaissances actuelles sur le sujet

- L’ostéosynthèse par mini plaque permet de réduire la durée de travail;
- Une ablation du matériel non systématique;
- Absence de confection d’attelle.

### Contribution de notre étude à la connaissance

- Nous pensons que l’ostéosynthèse par miniplaque reste la technique de référence dans le traitement des fractures déplacées des phalanges et des métacarpes.
